# Transcatheter aortic valve implantation *versus* surgical aortic valve replacement for treatment of severe aortic stenosis: comparison of results from randomized controlled trials and real-world data

**DOI:** 10.21470/1678-9741-2019-0288

**Published:** 2020

**Authors:** Dandan Wang, Litao Huang, Yuhui Zhang, Zeyi Cheng, Xin Zhang, Pengwei Ren, Qi Hong, Deying Kang

**Affiliations:** 1 Department of Evidence-based Medicine and Clinical Epidemiology, West China Hospital, Sichuan University, Chengdu, Sichuan, China.; 2 Department of Cardiovascular Surgery, West China Hospital, Sichuan University, Chengdu, Sichuan, China.; 3 Department of Integrated Traditional Chinese and Western Medicine, West China Hospital, Sichuan University.; 4 Clinical Research Center for Respiratory Diseases, West China Hospital, Sichuan University, Chengdu, Sichuan, China.

**Keywords:** Aortic Valve Stenosis, Transcatheter Aortic Valve Replacement, Risk, Randomized Controlled Trials as Topic, Meta-Analysis

## Abstract

**Objective:**

Results from randomized controlled trials (RCTs) and real-world study (RWS) appear to be discordant. We aimed to investigate whether data derived from RCTs and RWS evaluating long-term all-cause mortality of transcatheter aortic valve implantation (TAVI) *versus* surgical aortic valve replacement (SAVR) in patients with severe aortic stenosis (AS) were in agreement.

**Methods:**

RCTs or RWS comparing TAVI and SAVR, reporting longterm (≥2-year follow-up) all-cause mortality, were identified. We also carried out subgroup analyses to access the effect in different subgroups. A pre-designated data extraction form including 5 domains and 26 items was used to explore the relationship between RCTs and RWS. Mortality and effect in different subgroups were evaluated using random-effects meta-analyses.

**Results:**

Five RCTs (5421 participants, TAVI: 2759, SAVR: 2662) and 33 RWS (20839 participants; TAVI: 6585, SAVR: 14254) were identified. Pooled RCT analysis showed no difference in all-cause mortality between TAVI and SAVR (HR=0.97, 95% CI: 0.88-1.07; *P*=0.55). In RWS, TAVI was associated with an increased risk of allcause mortality (HR=1.46, 95% CI: 1.26-1.69; *P*<0.001) compared to SAVR.

**Conclusion:**

These results highlight the inconsistencies between RCTs and RWS in assessing long-term all-cause mortality in the treatment of AS using TAVI or SAVR, which may be caused by interactions of clinical characteristics or study design. RCTs as well as RWS are both developing and improving; the advantages of one kind of design, measurement and evaluation can and should be thoughtfully referred to the other.

**Table:** 

Abbreviations, acronyms & symbols				
**AS'**	**= Aortic stenosis**	** **	**RCTs**	**= Randomized controlled trials**
**AVR**	**= Aortic valve replacement**		**RWS**	**= Real-world study**
**CABG**	**= Coronary artery bypass grafting**		**RR**	**= Risk ratio**
**CENTRAL**	**= Cochrane Central Register of Controlled Trials**		**SAVR**	**= Surgical aortic valve replacement**
**CI**	**= Confidence intervals**		**STS-PROM**	**= Society of Thoracic Surgeons Predicted Risk of Mortality**
**CPB**	**= Cardiopulmonary bypass **		**SURTAVI**	**= Surgical Replacement and Transcatheter Aortic Valve Implantation**
**HR**	**= Hazard ratio**		**TAo**	**= Transaortic**
**NOTION**	**= Nordic Aortic Valve Intervention**		**TAP**	**= Transapical**
**NOS**	**= Newcastle-Ottawa Scale**		**TAVI**	**= Transcatheter aortic valve implantation**
**PARTNER**	**= Placement of Aortic Transcatheter Valve**		**TF**	**= Transfemoral**
**PICT**	**= Patients, intervention, control, time**			

## INTRODUCTION

Aortic stenosis (AS) is a common condition among elderly individuals and the prevalence of this disease increases with age, from 0.2% for ages 50-59 years to up to 10% for ages 80-89 years^[1-2]^. Without aortic valve replacement (AVR), symptomatic AS results in a life expectancy of <3 years^[3]^. The standard of treatment for patients with symptomatic severe AS is valve replacement, through surgical aortic valve replacement (SAVR) or by transcatheter aortic valve implantation (TAVI). So far, several randomized controlled trials (RCTs) comparing the two procedures have been conducted focusing on patients with high and low surgical risk. Three trials (Nordic Aortic Valve Intervention [NOTION]^[4]^, Placement of Aortic Transcatheter Valve [PARTNER 1]^[5]^ and CoreValve US High Risk Pivotal Trial [US CoreValve]^[6]^) have obtained 5-year follow-up results. RCTs confer the least biased estimates of treatment effects; their strict inclusion and exclusion criteria allow for recruiting participants most likely to benefit from an intervention. However, RCTs do not necessarily reflect real-world settings. Studies have shown that approximately 80% (82.2% in NOTION and 77.5% in PARTNER 1) of screened patients were excluded and not assigned to randomization^[7]^. On the other hand, real-world studies (RWS) may provide better generalizability to routine practice, as they often have less restrictive inclusion criteria, or tend to enrol "all comers". However, results of observational studies should always be interpreted cautiously because of greater potential bias of these studies. Comparison of these two study designs has indicated that their results may be conflicting^[8,9]^. This may be attributed to the complex interplaying of factors, such as the varied clinical characteristics (patients, intervention, control, time [PICT]) in addition to the study design. Therefore, this study compared data derived from RCTs and RWS on longterm all-cause mortality when treating AS with TAVI or SAVR and examined the clinical characteristics and clinical profiles that may underlie discrepancies.

## METHODS

We followed the reporting standards for systematic reviews and meta-analyses according to the PRISMA statements^[10]^. The concordance and reasons for discrepancies were further evaluated after conducting a meta-analysis.

### Search Strategy

EMBASE, MEDLINE, and the Cochrane Central Register of Controlled Trials (CENTRAL) were searched from inception to May 30, 2019. We used database-specific subject headings (e.g., MeSH terms) and free-text terms to search for potentially eligible studies (Supplementary Materials). We also searched ClinicalTrials.gov to identify additional relevant clinical studies.

### Inclusion Criteria

In both RCT and RWS settings, the intervention/exposure was transcatheter aortic valve replacement (TAVI group) and the control/non-exposure was surgical aortic valve replacement (SAVR group). TAVI was defined as the treatment of AS by displacing and functionally replacing the native valve with a bioprosthetic valve, delivered via a catheter, irrespective of whether the femoral artery (transfemoral [TF] placement), the transthoracic placement (transapical [TAp] or transaortic [TAo] access), or other access was used. SAVR was defined as the treatment of AS through median or minimal sternotomy under cardiopulmonary bypass (CPB). RCTs or RWS were included if they enrolled patients with severe AS, compared TAVI with SAVR, and reported long-term all-cause mortality (follow-up ≥2 years). For studies with duplicated data, we included only the report with the most informative and complete dataset.

### Assessment of Risk of Bias

Two researchers (DW, LH) assessed the methodological quality of the chosen studies. For RCTs, reviewers used the Cochrane risk of bias assessment tool^[11]^ to evaluate the following seven items: randomization sequence generation, allocation concealment, blinding of participants and personnel, blinding of outcome assessment, incomplete outcome data, selective reporting, and other bias. We defined other bias as company-sponsored trials and trials in which baseline characteristics differed between intervention groups. For RWS, the Newcastle-Ottawa Scale (NOS)^[12]^ was used to assess the risk of bias. Scores ≤6 were categorized as a high risk of bias, while >6 was considered a low risk of bias. The results were compared, and disagreements resolved by discussion.

### Data Extraction

Data derived from RCTs and RWS were extracted by two researchers (YZ, ZC) independently. A pre-designed data extraction form, including five domains and 26 items, was used to explore the relationship between data from RCTs and RWS. The number of events in each arm and the hazard ratio (HR) with the corresponding 95% confidence intervals (CIs) were extracted. HRs were given precedence over risk ratios (RRs), since they incorporate time-to-event data and allow censorship. When HRs with corresponding variance were not presented, we calculated them from Kaplan-Meier curve data or summary data using an HR calculation spreadsheet provided by Tierney et al.^[13]^ based on statistical methods reported by Parmar et al.^[14]^ Two reviewers independently completed all data extraction and disagreements were resolved through discussion or, if necessary, arbitration by a third reviewer (DK).

### Statistical Analysis

The main outcome was long-term all-cause mortality (over a follow-up period of at least 2 years). We used the random-effects model to pool the data and evaluated statistical heterogeneity between summary data using the I2 statistic. We performed subgroup analyses by the TAVI access route used for valve delivery (TF *vs.* TAp), sex (RCTs only), surgical risk (high- *vs.* nonhigh-risk patients), and type of TAVI (balloon-expandable *vs.* self-expandable); for RWS, geographic variation (Europe *vs.* North America *vs.* rest of the world) and whether the association of other procedures with SAVR (concomitant procedures *vs.* isolated SAVR) was used for stratification. Publication bias was assessed by visual inspection of funnel plot and statistical asymmetry was evaluated using the Begg's and Egger's test. Publication bias was assessed only when 10 or more studies were included.

Generally, descriptive statistics were used to summarize baseline variables in all groups. Data descriptions included frequencies and percentages for dichotomous data and mean with standard deviation or median with interquartile ranges for continuous data. Student’s t-test was used for comparison of means; when the data distribution was not normal, nonparametric tests were used (Mann-Whitney U test), and Pearson’s chi-square test was used for categorical comparisons. All analyses were performed using STATA version 14.0.

## RESULTS

### Studies Retrieved and Characteristics

The electronic search yielded 3564 unique citations (Figure 1). After full-text and reference screening, 38 studies, including five RCTs (NOTION, PARTNER 1A, PARTNER 2A, SURTAVI [Surgical Replacement and Transcatheter Aortic Valve Implantation], and US CoreValve) and 33 RWS, met the eligibility criteria (Details of included studies were presented in the Supplement).

**Fig. 1:**
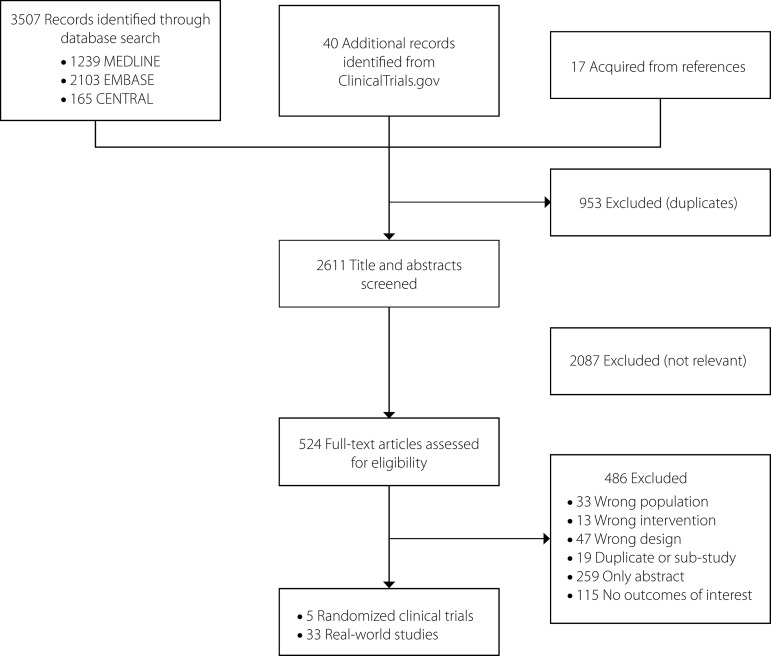
Flow chart of literature search and study selection.

The results of risk of bias assessment for the 38 included studies are presented in Figures 1 and 2 in the Supplement. There were three RCTs considered unclear in the item of allocation concealment, and two industry-funded trials were graded as having a high risk of bias based on other bias. No other bias was deemed present. Eighteen (54.5%) RWS were considered to have a high risk of bias and 15 (45.5%) to have low risk of bias, based on the NOS. Our funnel plot and statistical test demonstrated no evidence of publication bias (Begg's test: *P*=0.49; Egger's test: *P*=0.77) in RWS (Figure 2). However, the funnel plot interpretation for RCTs was not reported for the number of trials was five.

**Fig. 2:**
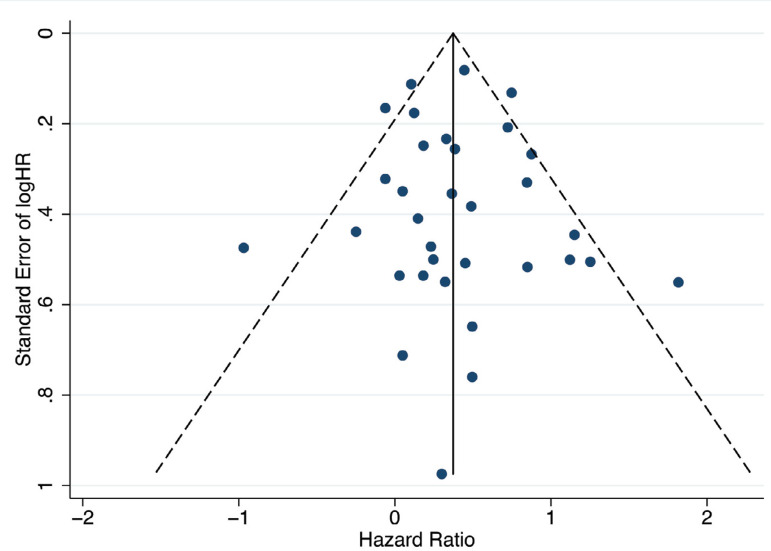
Funnel plot of the logarithm of the hazard ratio versus the standard error for each RWS.

The five RCTs included 5421 patients (TAVI: 2759, SAVR: 2662; mean age 81.5 [6.6] years; 2987 [55.1%] men), with one trial conducted in Europe and four in North America. The 33 RWS contained 20839 patients (TAVI: 6585, SAVR: 14254; mean age 77.5 [8.8] years; 11439 [54.9%] men), with 23 studies performed in Europe, four in North America, and six in the rest of the world. All studies were published between 2012 and 2019.

### All-Cause Mortality

Figures 3 and 4 present the metaanalysis results of ≥2-year mortality for RCTs and RWS, respectively. No significant differences were found in RCTs (HR: 0.97, 95% CI: 0.88-1.07, *P* for effect=0.55; I2=0%), while meta-analysis of RWS showed that all-cause mortality was significantly higher in patients treated with TAVI compared to SAVR (HR: 1.46, 95% CI: 1.26-1.69, *P* for effect <0.001; I2=48.9%).

**Fig. 3:**
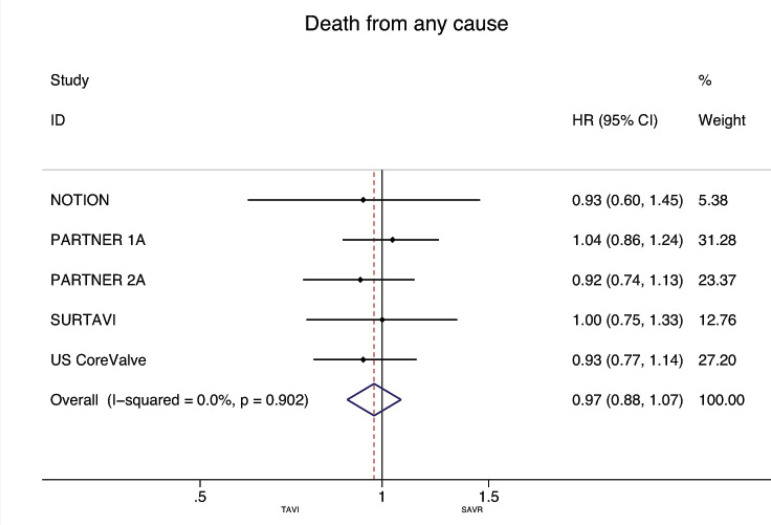
Forest plot of hazard ratios for ≥2-year all-cause mortality of RCTs. CI=confidence interval; HR=hazard ratios; NOTION=Nordic Aortic Valve Intervention; PARTNER=Placement of Aortic Transcatheter Valves; SAVR=surgical aortic valve replacement; SURTAVI=Surgical Replacement and Transcatheter Aortic Valve Implantation; TAVI=transcatheter aortic valve implantation; US CoreValve=CoreValve US High Risk Pivotal trial

**Fig. 4:**
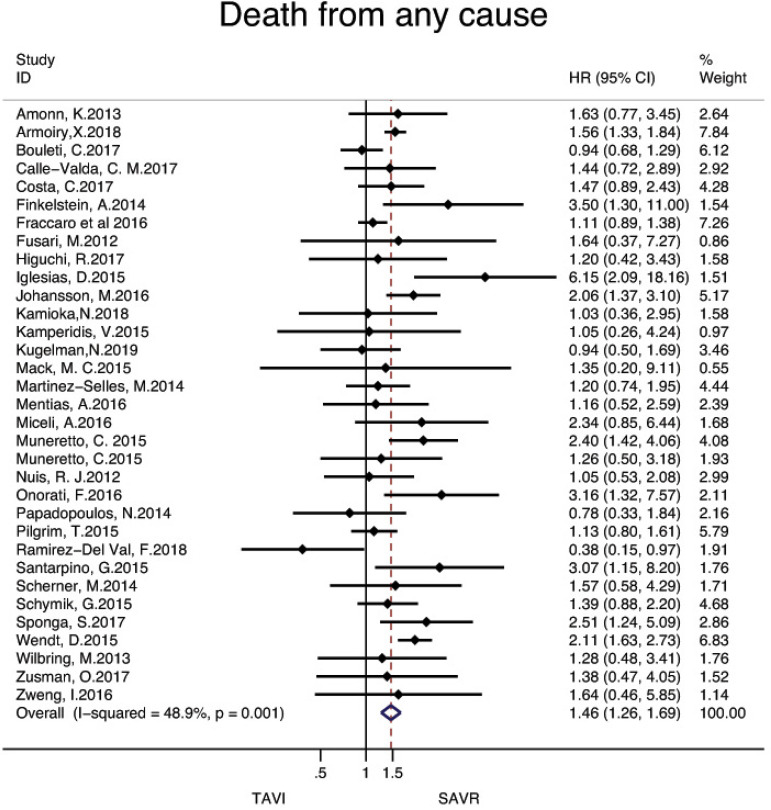
Forest plot of hazard ratios for ≥ 2-year all-cause mortality of RWS. CI=confidence interval; HR=hazard ratios; SAVR=surgical aortic valve replacement; TAVI=transcatheter aortic valve implantation

### Subgroup Analyses for Death from Any Cause

Stratified meta-analyses for death from any cause within the RCTs and RWS were performed in terms of access route (TF *vs.* TAp), geographic variations (RWS only), concomitant procedures with SAVR (RWS only), sex (RCTs only), surgical risk of participants (high *vs.* non-high-risk), and type of TAVI heart valve system (balloon-expandable *vs.* self-expandable) (Figures 5 and 6).

**Fig. 5:**
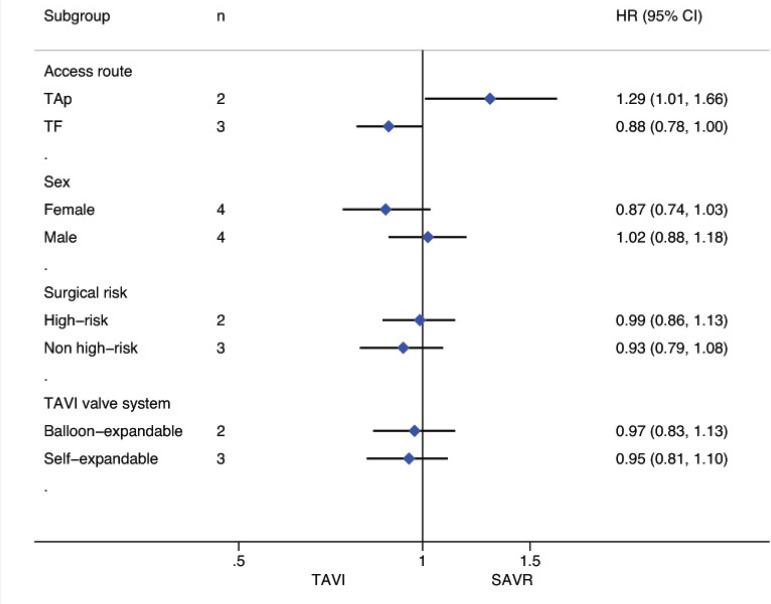
Subgroup analyses for death from any cause in RCTs. CI=confidence interval; HR=hazard ratios; SAVR=surgical aortic valve replacement; TAp=transapical; TAVI=transcatheter aortic valve implantation; TF=transfemoral

**Fig. 6:**
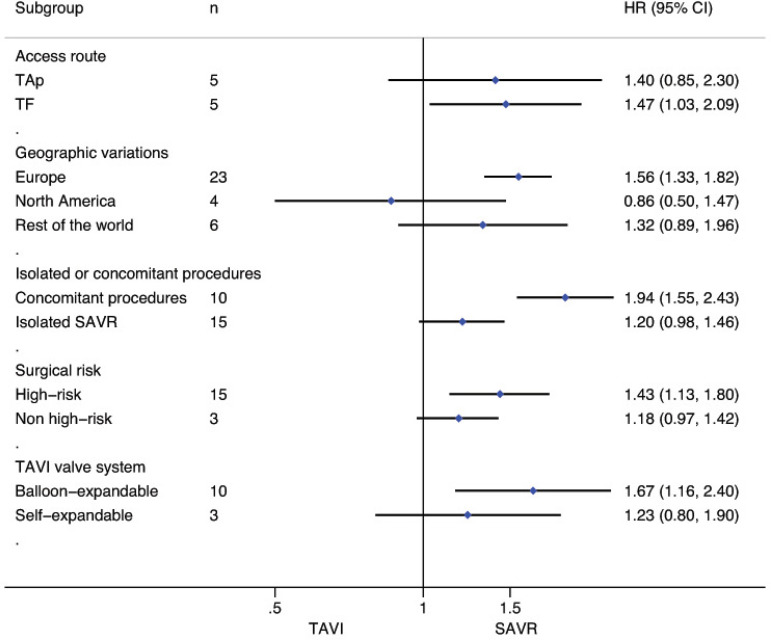
Subgroup analyses for death from any cause in RWS. CI=confidence interval; HR=hazard ratios; SAVR=surgical aortic valve replacement; TAp=transapical; TAVI=transcatheter aortic valve implantation; TF=transfemoral

For the RCTs, meta-analysis showed a survival benefit of participants distributed to TAVI through the TF route during follow-up compared to patients with SAVR (HR: 0.88, 95% CI: 0.78-1.00; *P*=0.05), while this result in RWS was the opposite (HR 1.47, 95% CI 1.032.09; *P*=0.03). SAVR had a survival benefit over TAVI among patients chosen for TAp access (HR: 1.29, 95% CI: 1.01-1.66; *P*=0.04) in RCTs, but not for the RWS group (HR 1.40, 95% CI 0.85-2.30; *P*=0.19).

Subgroup analyses of the surgical risk (high-risk *vs*. non-high-risk) as well as the TAVI system (balloon-expandable *vs.* selfexpandable) used showed no statistically significant difference in RCTs; while in RWS, SAVR was favoured over TAVI when used in high-surgical risk patients (HR: 1.43, 95% CI: 1.13-1.80; *P*=0.003), and when using a balloon-expandable TAVI system (HR: 1.67, 95% CI: 1.16-2.40; *P*=0.006).

We found no statistically significant difference between the techniques among female participants (HR: 0.87, 95% CI: 0.741.03, *P*=0.11) as well as among male patients (HR: 1.02, 95% CI: 0.88-1.18; *P*=0.78) in the RCT group. In RWS, other procedures in conjunction with SAVR differed significantly between the two groups, favouring SAVR (HR: 1.94, 95% CI: 1.55-2.43; *P*<0.001), but these effect was not observed in isolated SAVR (HR: 1.20, 95% CI: 0.98-1.46; *P*=0.08). A survival benefit was found for participants who underwent SAVR among the trials conducted in Europe (HR: 1.56, 95% CI: 1.33-1.82; *P*<0.001), but not for the trials performed in North America as well as in the rest of the world (HR: 0.86, 95% CI: 0.50-1.47; *P*=0.57; HR: 1.32, 95% CI: 0.89-1.96; *P*=0.16, respectively) (Figures 3-11 in the Supplement).

### Clinical Characteristics

We explored the concordance of data between RCTs and cohort studies to find the causes of discordance. The details of PICT and other important characteristics are summarized in Table 1. Compared with RWS, patients enrolled in RCTs were on average 4 years older (81.5±6.6 *vs.* 77.5±8.7), had more comorbidities and a lower Society of Thoracic Surgeons Predicted Risk of Mortality (STS-PROM) score (6.3±3.3% *vs.* 7.4±5.3%).

**Table 1:** Characteristics of RCTs and RWS.

Variables	RCTs (n=5)	RWS (n=33)	*P*-value
**Patients' characteristics**			
Age	81.5±6.6	77.5±8.7	<0.001^a^
Males	2987 (55.1)	11439 (54.9)	0.78^b^
STS-PROM	6.3±3.3	7.4±5.3	<0.001^a^
Diabetes mellitus	1654 (35.0)	4311 (22.2)	<0.001^b^
Chronic kidney disease	262 (7.0)	1690 (15.4)	<0.001^b^
Cerebrovascular disease	870 (28.9)	870 (8.4)	<0.001^b^
Prior PCI	1427 (26.3)	1161 (13.7)	<0.001^b^
Prior CABG	1302 (24.0)	785 (12.3)	<0.001^b^
Prior pacemaker	720 (13.3)	140 (6.9)	<0.001^b^
Peripheral vascular disease	1736 (32.0)	1495 (13.6)	<0.001^b^
Known AF/atrial flutter	1678 (31.0)	868 (26.0)	<0.001^b^
**TAVI's characteristics**			
**Access site**			<0.001^b^
TF	2358 (85.5)	1775 (63.5)	
Tap	313 (11.3)	992 (35.5)	
Others	87 (3.2)	30 (1.0)	
**TAVI valve system**			<0.001^b^
Self-expandable valve	1402 (50.8)	1576 (45.4)	
Balloon-expandable valve	1359 (49.2)	1895 (54.6)	
Edwards SAPIEN	348 (25.6)	859 (53.6)	
Edwards SAPIEN XT	1011 (74.4)	739 (46.1)	
Edwards SAPIEN 3	0	6 (0.4)	
**Procedure time (min)**	73.2±59.0	125.6±68.5	<0.001^a^
**Isolated or concomitant procedures**			<0.001^b^
Isolated TAVI	3177 (96.7)	2218 (92.6)	
Concomitant PCI	110 (3.3)	176 (7.4)	
**SAVR's characteristics**			
**Invasive approach**			<0.001^b^
Conventional approach	737 (91.4)	7319 (96.8)	
Minimally invasive approach	69 (8.6)	239 (3.2)	
**Valve system**			<0.001^b^
Biological prostheses	1597 (99.7)	2491 (97.1)	
Mechanical prostheses	5 (0.3)	75 (2.9)	
**Procedure time (min)**	210.6±68.4	269.1±119.0	<0.001^a^
**CPB time (min)**	99.7±41.5	111.7±65.3	<0.001^a^
**Aortic cross-clamp time (min)**	74.0±30.2	71.5±41.7	0.07^a^
**Isolated or concomitant procedures**			<0.001^b^
Isolated SAVR	4301 (87.2)	1842 (82.5)	
Concomitant procedures	634 (12.8)	392 (17.5)	
**Follow-up time (months)**	NA	23.1 (16.4, 38.8)	
**Other profiles**			
**Register**			<0.001^c^
Yes	5 (100.0)	5 (15.2)	
No	0	28 (84.8%)	
**Centres within studies**			<0.001^c^
Single centre	0	27 (81.8)	
Multicentre	5 (100.0)	6 (18.2)	
Centres per multicentre study	43.4 (3, 87)	28.8 (2, 93)	NA
Participants per multicentre study	1984	711	NA
Participants per centre	25	29	NA
**Conflict of interest**			<0.001^c^
Yes	5 (100.0)	15 (45.5)	
No	0	18 (54.5%)	
**Funding**			<0.001^c^
Industry-funded	4 (80.0%)	1 (3.0%)	
Government-funded	1 (20.0%)	2 (6.1%)	
Non-funded	0	30 (90.9%)	

Data are mean ± SD, median (interquartile range), or number (%) aStudent's t-test; bChi-square test; cFisher's exact test. AF=atrial fibrillation; CABG=coronary artery bypass grafting; CPB=cardiopulmonary bypass; NA=not applicable; PCI=percutaneous coronary intervention; STS-PROM=Society of Thoracic Surgeons Predicted Risk of Mortality; TAp=transapical; TF=transfemoral

In the TAVI (intervention/exposure) group, patients within RCTs were primarily assigned to the TF approach (2358/2758, 85.5%) and the total procedure time was shorter than that in RWS (73.2±59.0 *vs.* 125.6±68.5 minutes; *P*<0.001). The two TAVI heart valve systems (self-expandable and balloon-expandable) were used nearly equally in RCTs, and the Edwards SAPIEN XT (1011/1359, 74.4%) was the maximum balloon-expandable TAVI heart valve system; however, RWS used more balloonexpandable valves (1895/3471, 54.6%), in particular Edwards SAPIEN (859/1895, 53.6%). The proportion of isolated TAVI in RWS was significantly higher than that of RCT (96.7% *vs.* 92.6%).

Regarding SAVR (control/non-exposure), more minimally invasive approaches as well as more bio-prosthetic aortic valves were used in RCTs compared with RWS. A significantly shorter total procedure time (210.6±68.4 *vs.* 269.1±119.0 minutes) and total CPB time (99.7±41.5 *vs.* 111.7±65.3 minutes) were observed in RCTs compared with RWS, but this did not apply to total aortic cross-clamp time (74.0±30.2 *vs.* 71.5±41.7 minutes). The proportion of isolated SAVR was higher in RWS group rather than in RCT group (87.2% *vs.* 82.5%).

The median follow-up time in cohort studies was 23.1 months (interquartile range: 16.4 to 38.8 months). Only two RCTs presented this data (median follow-up time: 35.5 and 37.7 months, respectively).

All RCTs (5/5, 100%), but only 5/33 (15.2%) RWS were registered on ClinicalTrials.gov; more RCTs than RWS were multicentre studies (5/5, *vs.* 6/33, *P*<0.001). Eleven single-centre RWS were performed at university-affiliated hospitals, six at cardiovascular centres, one at a clinic, and the remaining eight studies failed to report this information. All RCTs presented conflict of interest disclosures, compared to only 45.5% of RWS.

## DISCUSSION

Long-term all-cause mortality when treating AS by TAVI or SAVR differed between RCTs and RWS. The discordance resulted not only from different study designs, but also heterogeneous clinical characteristics between RCTs and RWS. Compared with RWS, patients in RCTs were 4 years older, had more comorbidities, and had lower STS-PROM scores. The arterial route of TAVI access within RCTs was primarily TF and the mean total procedure time was markedly shorter; more patients in RCTs received newer generation TAVI balloon-expandable devices. The total SAVR procedure time as well as CPB time were markedly shorter in RCTs than in RWS. The RWS group had a higher percentage of isolated procedures (both isolated SAVR and isolated TAVI) than that in RCT group.

Patients undergoing TAVI in RCTs were older (81.6±6.6 years) and had a longer life expectancy than that in the US (79.1, 95% CI: 79.0-79.1)^[15]^. Non-valvular causes of death during the long-term follow-up may mask the surgical effect, which can be more significant in RCTs. In RWS, there is an inherent and practical bias in treatment selection, where physicians tend to select TAVI rather than SAVR for patients with a shorter life expectancy. While SAVR remains the standard treatment, several studies have demonstrated that 30% to 60% of patients with severe symptomatic AS are denied or not referred for surgery, leading to off-label use of TAVI^[16]^. An incomplete or poor risk-adjustment RWS may affect the results. For example, one research study that adopted several different adjustment methods to examine the effect of TAVI compared with SAVR found that TAVI could accordingly be associated with either substantial benefits or harms^[17]^. Factors such as the impact of the initial learning curve and the different algorithms for patient selection and perioperative curve may account for the discordancy in all-cause mortality with TAVI compared with SAVR in the RWS population *versus* the RCT population.

A survival benefit has been found for patients randomized to TAVI through TF access in RCTs. TF-TAVI is associated with higher rates of major vascular damage and those with more comorbidities are generally not eligible for this approach, particularly those with peripheral vascular disease^[18]^. While this result was the opposite in RWS, in this subgroup, an article^[19]^ had a significantly higher Charlson comorbidity index (an indicator of coexisting conditions) for TAVI, which meant patients who underwent TAVI had more complications than those in SAVR. We detected a detrimental effect associated with TAVI over SAVR when a balloon-expandable bioprosthesis (mainly SAPIEN) was used in RWS, while in RCTs, which mentioned more newer generation devices (SAPIEN XT), similar survival was achieved with TAVI and SAVR. The heart valve system used changed with time; for example, SAPIEN was replaced by SAPIEN XT and more recently SAPIEN 3 and Core Valve have been replaced by Evolute and Evolute R, respectively. It is reasonable to expect that newer generation devices might lead to better long-term outcomes.

Two included RWS tended to favour TAVI^[20,21]^; they were performed in the setting of "prior cardiac surgery (mostly coronary artery bypass grafting [CABG])" and "porcelain aorta", where surgical AVR is particularly challenging. This result is consistent with the recommendations from existing guidelines that suggest patient frailty, and conditions such as porcelain aorta, history of chest radiation, or patent coronary bypass grafts may render patients less suitable for SAVR^[22]^.

Our observation that TAVI was associated with worse longterm overall survival than SAVR in RWS was supported by the findings from a meta-analysis that involved 4197 patients with severe AS. Takagi et al.^[7]^. reported that the pooled results of 14 RWS with a propensity score analysis showed TAVI to be inferior to SAVR in 3-year overall survival. Another meta-analysis^[23]^ including four RCTs found TAVI was superior to SAVR in 2-year survival (HR: 0.87, 95% CI: 0.76-0.99; *P*=0.038). Compared with these studies, our study included both RWS and RCTs, and quantitatively compared the differences between the two research approaches by a thorough comparison, which makes our results more objective and convincing.

This study had several limitations. First, cohort studies were selected to represent the real-world situation, but their inherent limitations may lead to inaccurate results^[24,25]^. We attempted to minimize these limitations by performing a strict quality assessment, but poor reporting quality of the included studies did not allow definitive judgments about risk of bias in all domains. Second, this study concentrated on long-term all-cause mortality because it can provide non-biased results. Other important outcomes might yield additional clinical insights, and thus further research is required. Third, given the high cost of TAVI as well as SAVR, almost all 38 studies were performed in developed countries; therefore, these results may not be generalizable to developing countries. The number of multicentre, international studies was actually small, and we were unable to find large-scale and carefully conducted nationwide registry studies that may provide an objective realworld conclusion with robust risk adjustment.

## CONCLUSION

These results highlight the inconsistencies between RCTs and RWS in assessing long-term all-cause mortality when treating AS using TAVI or SAVR, which may be caused by interactions of clinical characteristics or study design. RCTs as well as RWS are both developing and improving; the advantages of one kind of design, measurement and evaluation can and should be thoughtfully referred to the other.

**Table:** 

Authors' roles & responsibilities	
DW	Substantial contributions to the conception or design of the work; or the acquisition, analysis or interpretation of data for the work; drafting the work or revising it critically for important intellectual content; final approval of the version to be published
LH	Substantial contributions to the conception or design of the work; or the acquisition, analysis or interpretation of data for the work; final approval of the version to be published
YZ	Substantial contributions to the conception or design of the work; or the acquisition, analysis or interpretation of data for the work; final approval of the version to be published
ZC	Substantial contributions to the conception or design of the work; or the acquisition, analysis or interpretation of data for the work; final approval of the version to be published
XZ	Substantial contributions to the conception or design of the work; or the acquisition, analysis or interpretation of data for the work; drafting the work or revising it critically for important intellectual content; final approval of the version to be published
PR	Substantial contributions to the conception or design of the work; or the acquisition, analysis or interpretation of data for the work; final approval of the version to be published
QH	Substantial contributions to the conception or design of the work; or the acquisition, analysis or interpretation of data for the work; drafting the work or revising it critically for important intellectual content; final approval of the version to be published
DK	Substantial contributions to the conception or design of the work; or the acquisition, analysis or interpretation of data for the work; drafting the work or revising it critically for important intellectual content; agreement to be accountable for all aspects of the work in ensuring that issues related to the accuracy or integrity of any part of the work are appropriately investigated and resolved; final approval of the version to be published

## SUPPLEMENTARY MATERIAL

**• Supplementary methods •**

**Supplementary Methods 1. Search Strategy in MEDLINE**

Ovid Technologies, Inc. Search limit to English language Database: Ovid MEDLINE(R) In-Process & Other Non-Indexed Citations and Ovid MEDLINE(R) <1946 to 2019 May 30 > Search Strategy:

1. exp Aortic Valve Stenosis/
2. aortic valve stenosis.mp.
3. aortic stenosis.mp.
4. exp Transcatheter Aortic Valve Replacement/
5. TAVR.af.
6. TAVI.af.
7. transcatheter.af.
8. tranfemoral.af.
9. transapical.af.
10. transaxillary.af.
11. transcatheter aortic valve implantation.mp.
12. surgical aortic valve repalcement.af.
13. surgical AVR.af.
14. SAVR.af.
15. 1 OR 2 OR 3
16. 4 OR 5 OR 6 OR 7 OR 8 9 OR 10 OR 11
17. 12 OR 13 OR 14
18. 15 AND 16 AND 17

**Supplementary Methods 2. Search Strategy in EMBASE.**

Ovid Technologies, Inc. Search limit to English language Database: EMBASE Classic + EMBASE <1947 to 2019 May 30 > Search Strategy:

1. exp aortic valve stenosis/
2. aortic valve stenosis.mp.
3. exp aortic stenosis/
4. aortic stenosis.mp.
5. exp transcatheter aortic valve implantation/
6. transcatheter aortic valve implantation.mp.
7. TAVR.af.
8. TAVI.af.
9. transcatheter.af.
10. transfemoral.af.
11. transapical.af.
12. transaxillary.af.
13. surgical aortic valve replacement.af.
14. surgical AVR.af.
15. SAVR.af.
16. 1 OR 2 OR 3 OR 4
17. 5 OR 6 OR 7 OR 8 OR 9 OR 10 OR 11 OR 12
18. 13 OR 14 OR 15
19. 16 AND 17 AND 18

**Supplementary Methods 3. Search Strategy in Cochrane Central**

Register of Controlled Trials (Ovid) (Search data: May 30, 2019)

1. exp aortic valve stenosis/
2. aortic stenosis.mp.
3. aortic valve stenosis.mp.
4. transcatheter aortic valve implantation.mp.
5. transcatheter aortic valve replacement.mp.
6. TAVR.af.
7. TAVI.af.
8. transcatheter.af.
9. transfemoral.af.
10. transapical.af.
11. transaxillary.af.
12. surgical aortic valve replacement.af.
13. SAVR.af.
14. surgical AVR.af.
15. 1 OR 2 OR 3
16. 4 OR 5 OR 6 OR 7 OR 8 OR 9 OR 10 OR 11
17. 12 OR 13 OR 14
18. 15 AND 16 AND 17

**Supplementary Methods 4. Search Strategy in ClinicalTrials.gov**

(Search data: May 30, 2019) " transcatheter aortic valve implantation" OR "TAVI" OR transcatheter OR transfemoral OR transapical OR transaxillary OR SAVR OR " surgical AVR" OR "surgical aortic valve replacement" | Studies With Results | Aortic Stenosis

**• Supplementary figures •**

**Fig. 1A supplement:**
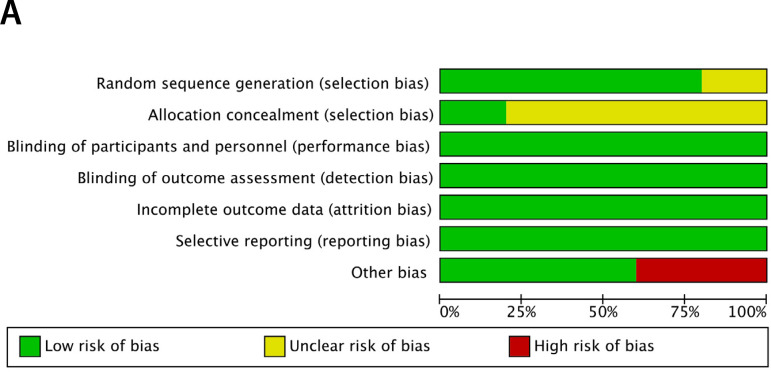
Risk of bias graph of included RCTs

**Fig. 1B supplement:**
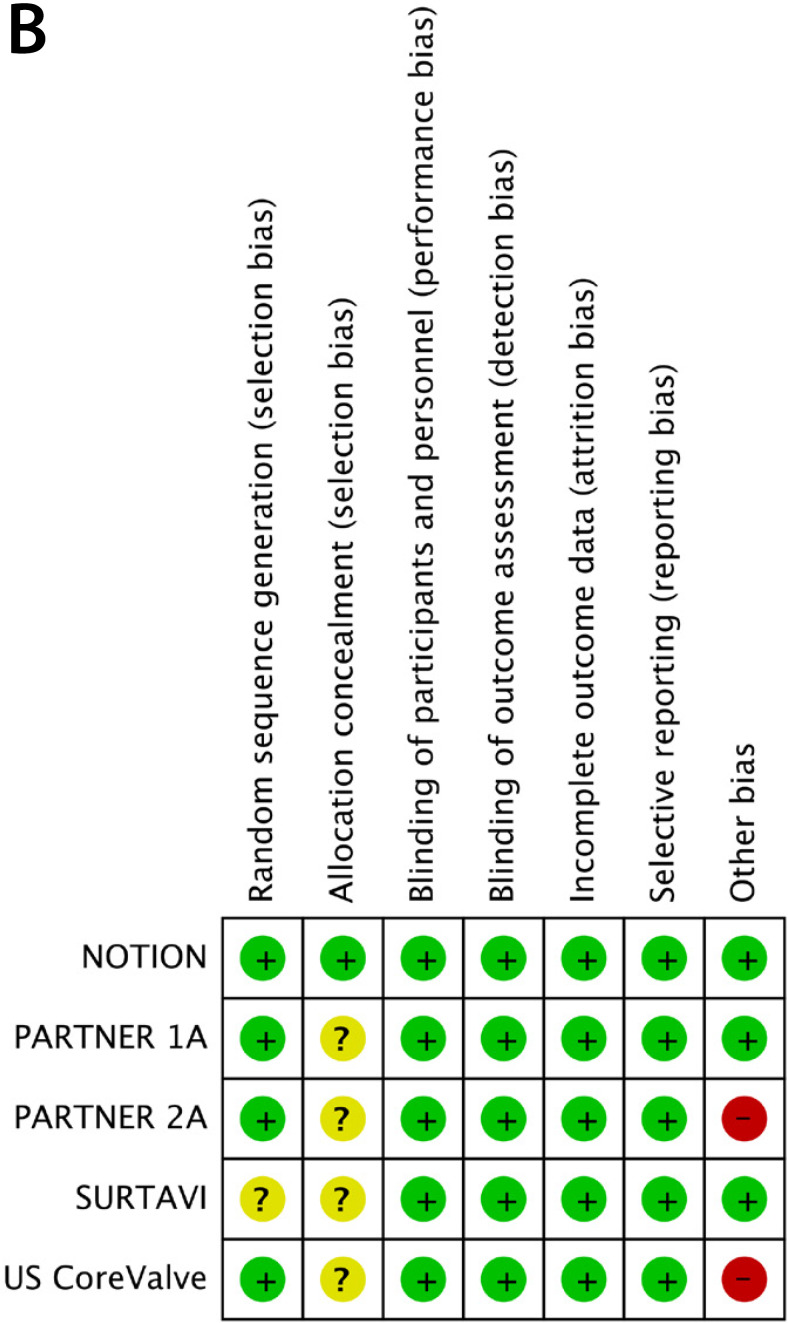
Risk of bias summary of included RCTs.

NOTION=the Nordic aortic valve intervention. PARTNER=the Placement of Aortic Transcatheter Valves; SURTAVI=the Surgical Replacement and Transcatheter Aortic Valve Implantation. US CoreValve=the CoreValve US High Risk Pivotal Trial

**Fig. 2 supplement:**
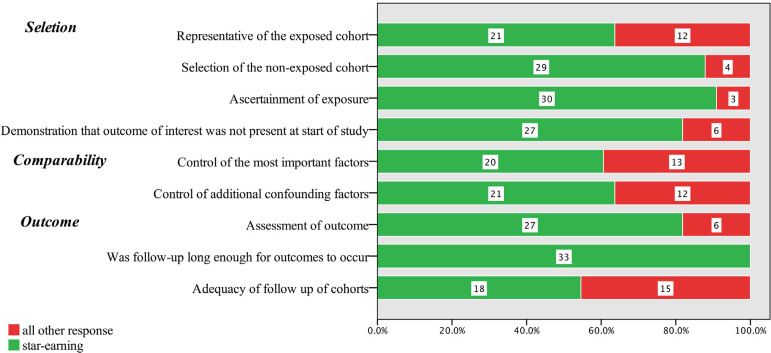
Risk of bias in the included real-world studies using the Newcastle-Ottawa Scale (NOS).

**Fig. 3 supplement:**
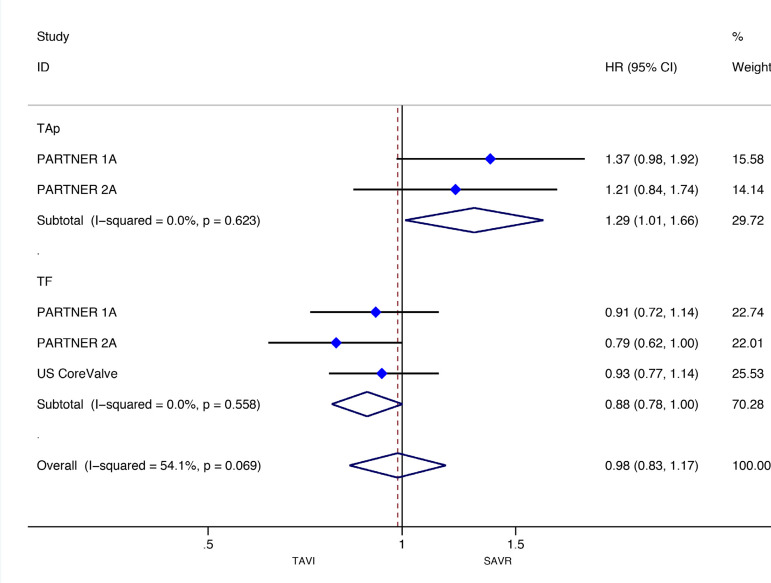
Subgroup analysis (access route) for death from any cause in RCTs. CI=confidence interval; HR=hazard ratio; PARTNER=the Placement of Aortic Transcatheter Valves; SAVR=surgical aortic valve replacement; TAp=transapical; TAVI=transcatheter aortic valve implantation; TF=transfemoral; US CoreValve=the CoreValve US High Risk Pivotal Trial

**Fig. 4 supplement:**
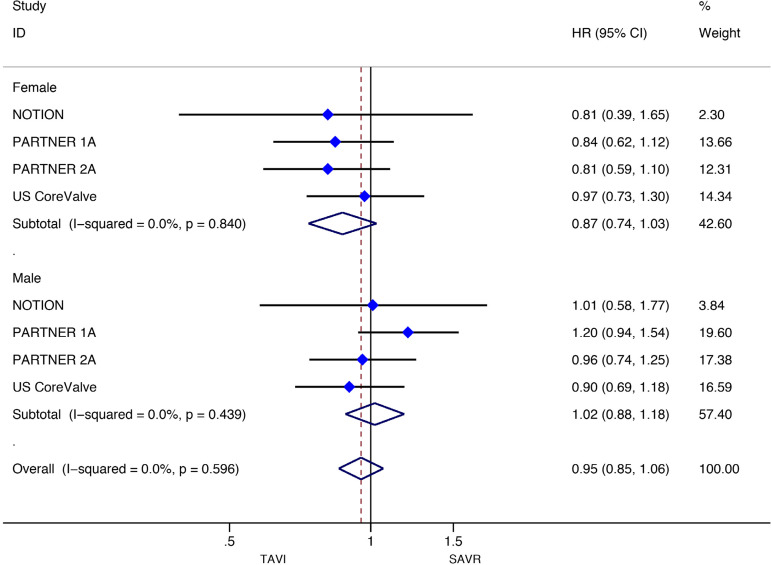
Subgroup analysis (sex) for death from any cause in RCTs. CI=confidence interval; HR=hazard ratio. NOTION=the Nordic aortic valve intervention; PARTNER= the Placement of Aortic Transcatheter Valves; SAVR=surgical aortic valve replacement; TAVI=transcatheter aortic valve implantation; US CoreValve= the CoreValve US High Risk Pivotal Trial

**Fig. 5 supplement:**
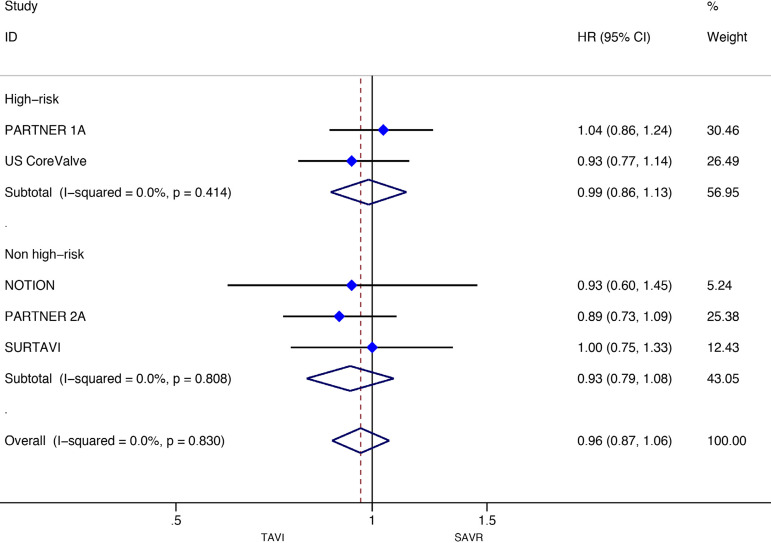
Subgroup analysis (surgical risk of participants) for death from any cause in RCTs. CI=confidence interval; HR=hazard ratio; NOTION=the Nordic aortic valve intervention; PARTNER=the Placement of Aortic Transcatheter Valves; SAVR=surgical aortic valve replacement; SURTAVI=the Surgical Replacement and Transcatheter Aortic Valve Implantation; TAVI= transcatheter aortic valve implantation; US CoreValve=the CoreValve US High Risk Pivotal Trial

**Fig. 6 supplement:**
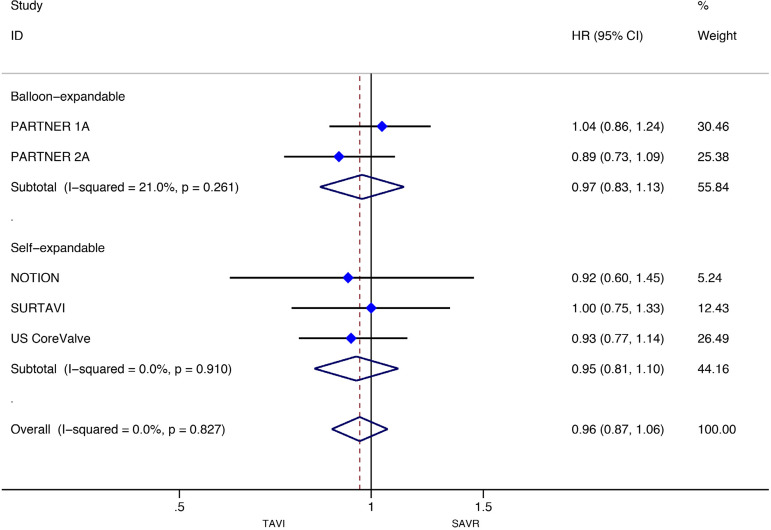
Subgroup analysis (TAVI heart valve system) for death from any cause in RCTs. CI=confidence interval; HR=hazard ratio; NOTION=the Nordic aortic valve intervention; PARTNER=the Placement of Aortic Transcatheter Valves; SAVR=surgical aortic valve replacement; SURTAVI=the surgical replacement and transcatheter aortic valve implantation; TAVI=transcatheter aortic valve implantation; US CoreValve=the CoreValve US High Risk Pivotal Trial

**Fig. 7 supplement:**
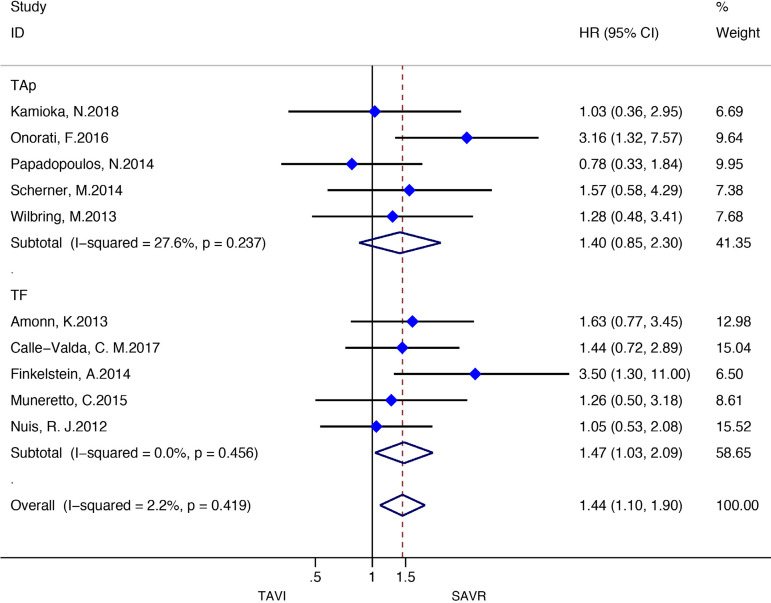
Subgroup analysis (access route) for death from any cause in RWS. CI=confidence interval; HR=hazard ratio; SAVR=surgical aortic valve replacement; TAp=transapical; TAVI=transcatheter aortic valve implantation; TF=transfemoral

**Fig. 8 supplement:**
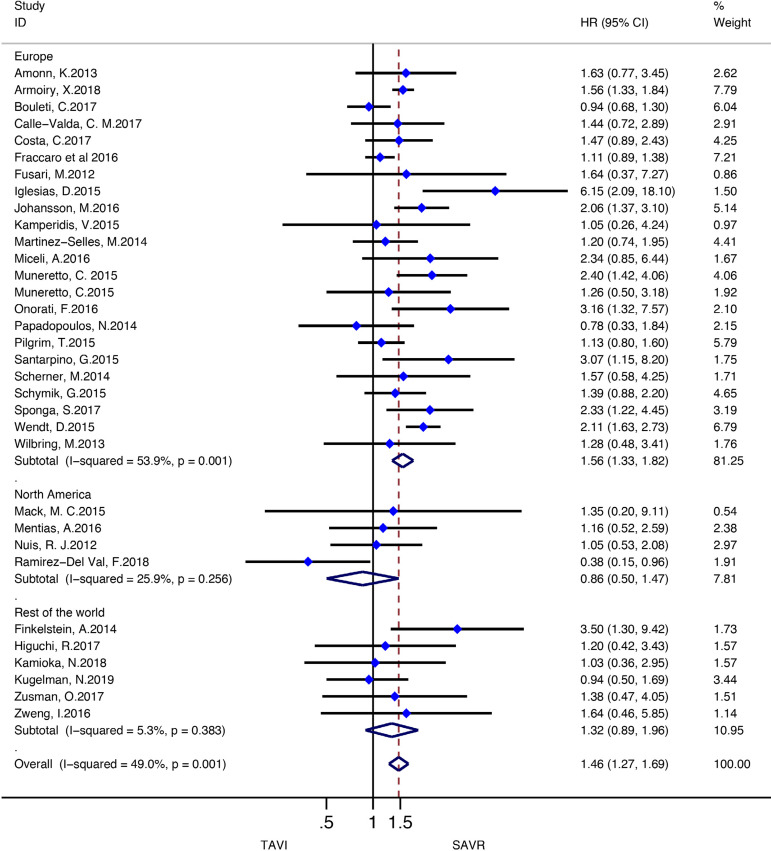
Subgroup analysis (geographic variations) for death from any cause in RWS. CI=confidence interval; HR=hazard ratio; SAVR=surgical aortic valve replacement; TAVI=transcatheter aortic valve implantation

**Fig. 9 supplement:**
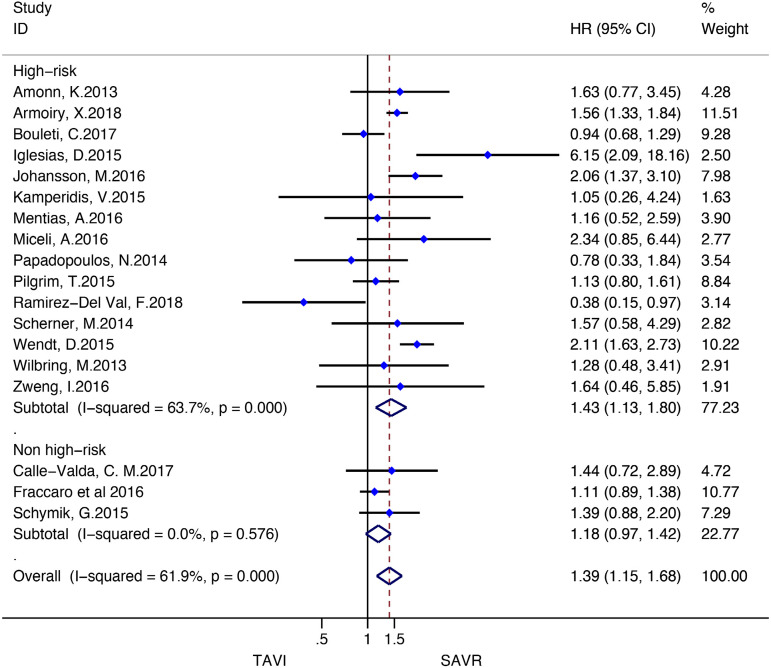
Subgroup analysis (surgical risk of participants) for death from any cause in RWS. CI=confidence interval; HR=hazard ratio; SAVR=surgical aortic valve replacement; TAVI=transcatheter aortic valve implantation

**Fig. 10 supplement:**
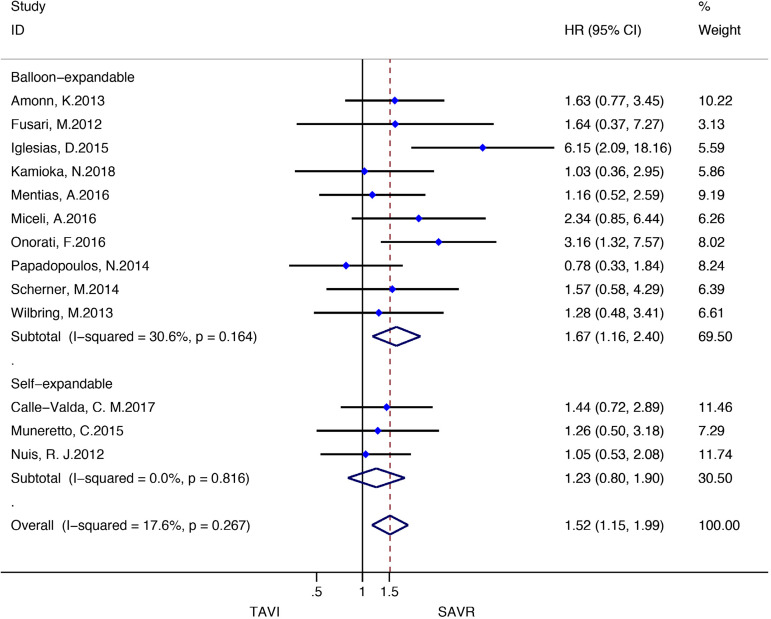
Subgroup analysis (TAVI heart valve system) for death from any cause in RWS. CI=confidence interval; HR=hazard ratio; SAVR=surgical aortic valve replacement; TAVI=transcatheter aortic valve implantation

**Fig. 11 supplement:**
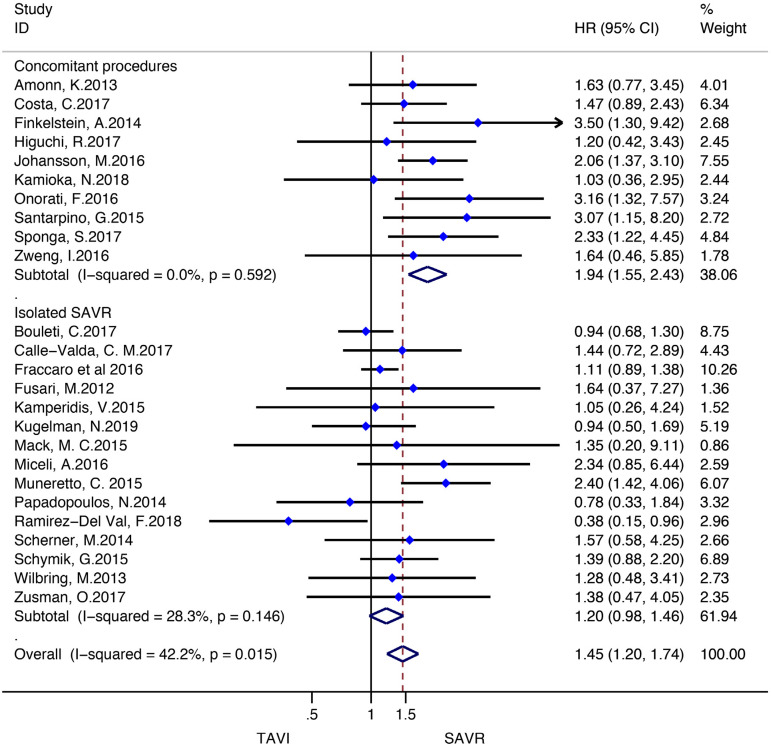
Subgroup analysis ( Isolated or concomitant procedures) for death from any cause in RWS. CI=confidence interval; HR=hazard ratio; SAVR=surgical aortic valve replacement; TAVI=transcatheter aortic valve implantation
